# Group physiotherapy compared to individual physiotherapy to treat urinary incontinence in aging women: study protocol for a randomized controlled trial

**DOI:** 10.1186/s13063-017-2261-4

**Published:** 2017-11-16

**Authors:** Chantale Dumoulin, Mélanie Morin, Marie-Hélène Mayrand, Michel Tousignant, Michal Abrahamowicz

**Affiliations:** 1 School of Rehabilitation, Faculty of Medicine, Université de Montréal, Research Centre of the Institut Universitaire de Gériatrie de Montréal, 4565 Queen Mary M-5816, Montreal, QC H3W 1W5 Canada; 2 School of Rehabilitation, Faculty of Medicine and Health Sciences, Université de Sherbrooke and Research Center of the Centre hospitalier universitaire de Sherbrooke (CHUS), Sherbrooke, QC Canada; 3 Department of Obstetrics and Gynecology and Social and Preventive Medicine, Université de Montréal and Research Center of the Centre Hospitalier de l’Université de Montréal, Montréal, QC Canada; 4 School of Rehabilitation, Faculty of Medicine and Health Sciences, Université de Sherbrooke and Research Center on Aging, Sherbrooke, QC Canada; 5 Department of Epidemiology, Biostatistics and Occupational Health, McGill University, Research Institute of the McGill University Health Centre, Montréal, QC Canada

**Keywords:** Urinary incontinence, Pelvic floor muscle training, Elderly women

## Abstract

**Background:**

Urinary incontinence (UI), one of the most prevalent health concerns confronting women aged over 60 years, affects up to 55% of older community-dwelling women—20–25% with severe symptoms. Clinical practice guidelines recommend individualized pelvic floor muscle training (PFMT) as a first-line treatment for stress or mixed UI in women, although lack of human and financial resources limits delivery of this first-line treatment. Preliminary data suggest that group-based treatments may provide the answer. To date, no adequately powered trials have evaluated the effectiveness or cost-effectiveness of group compared to individual PFMT for UI in older women. Given demographic projections, high prevalence of UI in older women, costly barriers, and group PFMT promising results, there is a clear need to rigorously compare the short- and long-term effectiveness and cost-effectiveness of group vs individual PFMT.

**Methods/Design:**

The study is designed as a non-inferiority randomized controlled trial, conducted in two facilities (Montreal and Sherbrooke) in the Canadian province of Quebec. Participants include 364 ambulatory, community-dwelling women, aged 60 years and older, with stress or mixed UI. Randomly assigned participants will follow a 12-week PFMT, either in one-on-one sessions or as part of a group, under the supervision of a physiotherapist. Blinded assessments at baseline, immediately post intervention, and at one year will include the seven-day bladder diary, the 24-h pad test, symptoms and quality of life questionnaires, adherence and self-efficacy questionnaire, pelvic floor muscle function, and cost assessments. Primary analysis will test our main hypothesis that group-based treatment is not inferior to individualized treatment with respect to the primary outcome: relative (%) reduction in the number of leakages.

**Discussion:**

Should this study find that a group-based approach is not less effective than individual PFMT, and more cost-effective, this trial will impact positively continence-care accessibility and warrant a change in clinical practice.

**Trial registration:**

ClinicalTrials.gov, NCT02039830. Registered on 12 December 2013; Study protocol version 2; 21 November 2013.

**Electronic supplementary material:**

The online version of this article (doi:10.1186/s13063-017-2261-4) contains supplementary material, which is available to authorized users.

## Background

Urinary incontinence (UI), one of the most prevalent health concerns confronting women aged 60 years and over, affects up to 55% of older community-dwelling women [1]—20–25% having severe symptoms (> 10 UI episodes/week) [1]. Recognized as a serious medical condition, UI is also a social issue, one that engenders shame and negative self-perception leading to reduced social interaction and physical activity [2–4]. It is associated with poor self-rated health, impaired emotional and psychological wellbeing, and impaired sexual relationships [3, 5, 6]. It doubles women’s risk of being admitted to a nursing home, independently of age or the presence of any other co-morbid conditions [7].

Clinical practice guidelines recommend individualized pelvic floor muscle training (PFMT) as a first-line treatment for stress or mixed UI in women (Level A evidence [8–12]); however, inadequate financial and human resources prevent delivery of individualized PFMT in many countries [13–15].

Evidence from two recent randomized controlled trials (RCTs) suggests that group PFMT is effective for treating stress and mixed UI in older women, resulting in high continence rates post-intervention compared to no treatment or bladder training [16, 17]. A group-delivery approach offers an effective way to overcome financial and human resource barriers; moreover, it has been shown to increase participant’s motivation, adherence, and UI self-management capabilities [18–22]. In recent years, the search for less costly forms of rehabilitative treatments has taken on a greater impetus and been subject to intense debate [8, 23, 24]. It is now recognized that for some pathological conditions, group rehabilitation approaches offer a viable solution, one that would permit better allocation of available economic resources—material and human [23]. Additionally, group intervention is already acknowledged, within the field of health promotion, as a powerful tool for promoting behavior modification: group sessions provide greater motivation (by reducing an individual’s sense of isolation) and a forum for providing information (particularly for those too timid to ask questions) and foster peer support and discussions: e.g. Weight Watchers [25]. Tackling UI through group sessions could also prove to be an effective means of educating and encouraging active self-management; therefore, it could have an impact in the longer term. [26, 27] To date, no studies have compared with adequate power the long-term effectiveness or cost-effectiveness of group PFMT to individualized PFMT for the treatment of UI in older women [18].

Given group PFMT’s promising results, there is a clear need to compare the short- and long-term effectiveness and cost-effectiveness of group vs individual PFMT, especially considering demographic projections, the high prevalence of UI in older women and the costly barriers to individualized PFMT. If we demonstrated that group-based treatment is not meaningfully less effective than individualized one-on-one treatment, and more cost-effective, group-based PFM training would be warranted as a first-line UI treatment.

### Rationale for a non-inferiority trial

A non-inferiority design was chosen because: (1) individual PFMT is the standard of care; (2) recent literature and our preliminary data on aging women suggest that group-based PFMT may be effective immediately post treatment and in the short term (less than six months); (3) there is potential for long-term benefits from group-based interventions resulting from increased peer-support and mutual self-help leading to increased compliance with treatment; (4) the anticipated lower cost; and (5) the potential to improve accessibility to care through a group approach (overcoming lack of human and financial resources). Should this study find that a group-based PFM training approach is not meaningfully less effective than individual PFM training, it would warrant a change in clinical practice.

### Objectives

The overall objective of the GROUP (Group Rehabilitation Or IndividUal Physiotherapy for Urinary Incontinence in Aging Women) trial is to determine if group-based PFMT for women aged 60 years and older with stress or mixed UI is not meaningfully less effective, sustainable, and affordable than the currently recommended individualized (one-on-one) PFMT.

The specific objectives are to compare the effectiveness of group-based PFMT vs individualized PFMT on:

1. the % reduction in the number of UI episodes, as measured by the seven-day diary [28], immediately post intervention, and at one year post randomization (primary outcome);
2. lower urinary track symptoms, level of distress, and quality-of-life impact, immediately post intervention and at one year post randomization. These secondary outcomes will be measured by, respectively, the seven-day bladder diary (number of urinary incontinence, number of micturition [28], the 24-h pad test (quantity of urine loss) [29], five modules of the International Consultation on Incontinence Questionnaire (ICIQ): the ICIQ-Urinary Incontinence short form (ICIQ-UI short form) [30], the ICIQ-Nocturia (ICIQ-N) [31], the ICIQ-Lower Urinary Tract Symptoms quality of life (ICIQ-LUTSquol) [32], the ICIQ-Vaginal symptoms (ICIQ-VS) [33], the ICIQ-Female Lower Urinary Tract Symptoms sex (ICIQ-FLUTSex) [34];
3. self-efficacy, immediately post intervention and at one year post randomization, as measured by the Geriatric Self Efficacy scale [35] and the Broom Self Efficacy questionnaire Part A [36];
4. impression of improvement and benefits, immediately post intervention and at one year post randomization as measured by the Patient Global Impression of Improvement (PGI-I) [37] and the Satisfaction and willingness to have another treatment (B&W) [17];
5. PFM strength, function, and morphometry as measured by the digital Oxford scale [38], pelvic floor dynamometry [39], transperineal US [40] immediately post intervention and at one year post randomization.

We will also compare the direct costs of group-based PFMT and individualized PFMT using the adapted Dowell–Bryant Incontinence Cost Index (DBICI) [41] immediately post intervention and at one year post randomization.

## Methods

### Trial design

**Fig. 1:**
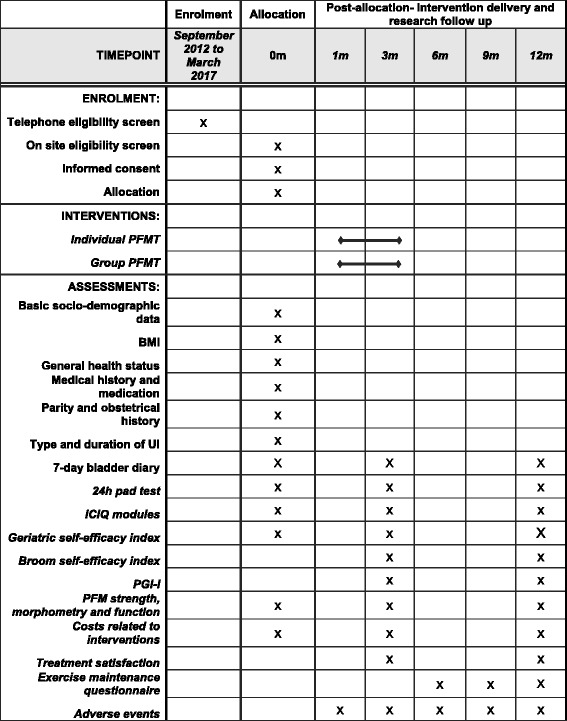
Schedule of enrolment, interventions, and assessments

The present study is a non-inferiority RCT involving two metropolitan areas in the province of Quebec, Canada: Montreal and Sherbrooke. This trial design is based on recommendations outlined in the “Research Methodology” chapter of the *International Consultation on Incontinence* book [42]. It comprises three parallel evaluations at, respectively, pre-intervention, post-intervention (1–2 weeks after treatment will have been completed), and one year post randomization, each involving structured interviews, questionnaires, bladder diary, pad tests, as well as physical and gynecological exams (Fig. 1).

### Research sites

The study is conducted at the research center of the Institut Universitaire de Gériatrie de Montréal and at the research center of the Centre Hospitalier de l’université de Sherbrooke.

### Participants

The target population is older women with stress UI and mixed UI. “Older” is defined as aged 60 years and over; as a cut-off age, this population of post-menopausal women have a UI profile distinctive from that of pre-menopausal or post-partum women [43]. Other RCTs on UI in older women also employ this cut-off age [17, 44]. There is no upper age limit as women as old as 98 years have been cured of UI through PFMT [45]. Participants are classified as incontinent if they report a weekly average of three or more episodes of involuntary urine loss during the preceding three months. This is a validated indicator of UI that has been used in cohort studies and RCTs on UI [16, 44]. Finally, type of UI is confirmed as a pattern of stress/mixed UI on the validated Questionnaire for Incontinence Diagnosis (QUID) [46].

### Recruitment

Women are being recruited from community advertisements, newspaper ads, the Research Center of the Institut Universitaire de Gériatrie de Montréal’s bank of participants, gynecology and urology clinics within the metropolis of the two study centers. In Montreal, these include the gynecology clinic at the Maisonneuve-Rosemont Hospital, and the CHUM gynecology clinics, the CLSC Lucille Teasdale, the CSSS Jeanne Mance. In Sherbrooke, this includes the urology and gynecology clinics at the Centre Hospitalier Universitaire de Sherbrooke. A poster explaining the research project is being displayed at each clinic. Interested candidates are invited to call the research coordinator.

Determination of eligibility is achieved in two steps: (1) telephone eligibility evaluation with a research assistant; and (2) on-site eligibility evaluation with the evaluator. Participant eligibility criteria are described in Table 1. These criteria ensure recruitment of a homogeneous sample of community-based older women with stress/mixed urinary incontinence signs and symptoms. Women excluded from the study are referred back to their healthcare provider to receive standard incontinence care.

**Table 1:** Inclusion and exclusion criteria

Inclusion criteria	
- Aged 60 years and over - Have stress or mixed urinary symptoms at least three times a week persisting for three months or more	
- Able to have a gynecological examination	
Exclusion criteria	
- Have a BMI ≥ 35; - Reduced mobility (not able to get around without the aid of a cane, crutches, or a walker) - Suffer from untreated chronic constipation - Experience important organ prolapse (POPQ > 2) - Received physiotherapy treatment for urinary incontinence in the past year - Have a surgery for incontinence or organ prolapse in the past year - Currently taking any medication for urinary incontinence (eligible if comfortable stopping the medication) or medications affecting skeletal muscles - Experiencing any leakage of stool or mucus - Have an active urinary or vaginal infection in last three months - Recent change in hormonal replacement - Any co-morbidities or risk factors interfering with the study (e.g. respiratory, cardiovascular, or memory problems, active cancer, diabetes, etc.)	

### Telephone eligibility evaluation (20 min)

Initial contact is over the phone. A research assistant briefly explains the project. Potential candidates are informed of the study’s objectives and procedures. If interested, they are screened for eligibility using a standardized telephone questionnaire developed and used in our previous studies. The UI type is determined by the QUID over the telephone; it is a six-item self-administered questionnaire developed to identify and differentiate UI types in women [46]. This instrument has shown good internal consistency and test–retest reliability, good content and criterion validity, and good specificity/sensitivity in women with UI [47]. Further, QUID is highly recommended for UI type classification by the International Consultation on Incontinence (Grade A) [47]. When women present with stress or mixed UI, the consent form (Additional file 1) is mailed out to the participant with the seven-day bladder diary [28, 47]; the diary is completed and brought to the evaluator at the onsite eligibility evaluation.

### Onsite eligibility evaluation (1 h)

Women are scheduled for onsite evaluations with a physiotherapist if they report at least three UI episodes on the seven-day bladder diary. After signing the consent form, the Mini-Mental State Examination [48] questionnaire is completed (excludes women with cognitive impairment) and a vaginal exam is conducted to identify any perineal pain or prolapse likely to interfere with either the evaluation or the intervention. Those not eligible for the present study are referred back to their healthcare provider. Eligible participants complete the evaluation and are randomized to either individualized or group PFMT.

### Interventions

The women in both groups receive the same 12-week PFMT, either in one-on-one sessions or as part of a group, under the direction of an experienced physiotherapist trained in pelvic floor rehabilitation. The choice of a 12-week PFMT approach is based on muscle physiology theory: strength-training programs show positive effect after 8–12 weeks [49]. In post-menopausal women, Gunnarsson et al. reported a significant improvement in strength and reduction in UI episodes after 12 weeks of training [50]. Both the one-on-one and group-based PFMT sessions are conducted once a week; participants are deemed to have successfully completed the program if they attended ten or more of the 12 sessions. Although there is no known side effect/complication related to PFMT other than possible discomfort following the intervention, any adverse event is monitored. All participants are instructed to contact research personnel should they experience any adverse event at any point during the study.

### PFMT sessions

For both groups, the weekly sessions last 1 h and include a 15-min educational period and a 45-min exercise component. The educational period covers the various functions of the PFMs, including pre-contraction and normal bladder control, and voiding parameters: fluids and fluid intake; toilet positions; and voiding dynamics. The exercise component includes PFM strength, endurance, and coordination exercises. Between PFM exercises, lower extremity strength, balance and functional exercises (dance) are performed. This approach is in line with our previous research findings, which indicate that mixed and stress UI older women have reduced PFM and lower-extremity strength and balance as compared to continent women [18, 51, 52]. It is also a component of PFMT programs designed for the elderly [16, 17]. The treatment protocol is divided into three phases allowing for gradual progression in treatment; that is, the gradual addition of increasingly difficult exercises in terms of exercise duration, repetition, and position. Each phase lasts four weeks. See Table 2 for monthly treatment’s exercise details.

**Table 2:** Monthly treatment’s exercise details

	Warm-up exercises	Pelvic floor exercises				Core exercises	Dance
		Knack^a^	Maximal contractions^a, b^	Fast contractions	Podiums^c^		
Weeks 1–4	Anterior and posterior pelvic tilts standing (4 reps), plantar flexion and dorsal flexion of the ankle (4 reps bilaterally)	(1 cough only) 3 reps	6 reps, 6-s hold	—	3 reps, 6 s/step	Pull belly button towards spine four-point kneeling (6 reps, hold 6 s), bridge (6 reps, 6 s hold)	3 beginner songs
Weeks 5–8	Simple rotation of the pelvis standing (4 reps), plantar flexion and dorsal flexion of the ankle (4 reps bilaterally)	(2 coughs) 3 reps	8 reps, 8-s hold	2 reps, 8 contractions	3 reps, 8 s/step	Pull belly button towards spine four-point kneeling + contraction of the PFM + lifting of the arm (8 reps, 8-s hold)	3 intermediate songs
Weeks 9–12	“8 shaped” rotation of the pelvis standing (4 reps), plantar flexion and dorsal flexion of the ankle (4 reps bilaterally)	(3 coughs) 3 reps	10 reps, 10-s hold	2 reps, 10 contractions	3 rep, 10 s/step	Pull belly button towards spine four-point kneeling + contraction of the PF+ lifting of one arm (8 reps, 8-s hold), contraction of PFM+ contraction transverse + bridge (6 reps, 8-s hold)	3 advanced songs

^a^The knack involves pre-contracting the PFM before and during a cough

^b^The maximal contractions: is done through different positions depending on the week

^c^Podium exercise (moderate-maximal-moderate contraction): can be inverted depending on the week

*Reps* repetitions, *s* seconds, *PFM* pelvic floor muscle

For the individual treatment group, the pelvic floor exercises: can be done with a biofeedback probe depending on the week

#### Individualized PFMT

The only specificity of this treatment group is that women participate in one-on-one, 1-h sessions with intra-vaginal electomyography (EMG) biofeedback under the supervision of an experienced physiotherapist. This treatment approach is in line with clinical practice in Canada [53].

#### Group-based PFMT

The only specificity for this treatment group is that women participate in groups of eight in a weekly 1-h session, under the supervision of an experienced physiotherapist. They are also offered up to three optional private (20 min) sessions with the physiotherapist leading their group (either before or after a group session) to ensure understanding and correct performance of a PFM contraction – as confirmed by vaginal digital palpation [17]. This treatment approach is consistent with Bo’s PFM exercise class [54] and our more recent pilot cohort studies indicating it is both feasible and effective intervention for UI in aging women [18, 55]. In case of slow recruitment, we have established a minimum group size of six participants in order to avoid delays for those assigned to group physiotherapy.

### Home PFMT program

Women in both groups are expected to perform PFM strengthening, endurance, and coordination exercises at home, five days a week, for the duration of the treatment [18, 22, 51, 55]. To support progression in the treatment, the home exercise program parallels the three phases in the treatment protocol with the gradual addition of increasingly difficult exercises every four weeks. To standardize home PFM exercises, each participant is given a PFM exercise diary describing the home PFMT exercises, in which they can record their adherence to the home program. Finally, all participants are asked to refrain from seeking other forms of treatment (such as medication or surgery) during the study.

### Standardization of treatment

Physiotherapists who deliver the interventions are extensively trained in standardized treatment protocols and rigorous procedures to conduct both individualized and group-based PFMT. Each physiotherapist participates in a 4-h training workshop, is given a written a treatment protocol with checklist, and is individually supervised for three to six treatments. Routinely, during the course of the study, all physiotherapists come together with the study team to ensure that consistency in the protocol is being maintained and to discuss concerns that may arise. For physiotherapist effect not to confound results, the same physiotherapist is conducting both individualized and group-based PFM training in a single location.

### Randomization/blinding

#### Sequence generation and allocation concealment

To prevent imbalance on important patient characteristics while ensuring equal sizes of the two trial arms, we used stratified randomization with random blocks within each stratum. Specifically, participant allocation is stratified by (1) center (Montreal and Sherbrooke); and then (2) within each center, by UI type (MUI and SUI). Within each of the four (center-by-UI type) resulting strata, the randomization sequence was generated through a computerized system, by a CRIUGM statistician (FG), before the trial to create random permutated blocks of varying sizes (4–6), making the particular sequences difficult to predict. Randomization process takes place after a participant’s initial evaluation and written consent. Randomization lists are then used, by an independent individual (CRIUGM IT service analyst), to assign eligible participants to one of two trial arms.

#### Intervention allocation

A research assistant (one in each center) communicates with the CRIUGM IT service analyst to get the next sequential allocation and inform the participants as to which treatment they have been allocated and, in addition, organize the logistics of the PFMT intervention.

#### Blinding

Investigators, data analysts, and physiotherapists in charge of outcome evaluations remain blinded to the individual participants trial group allocation. Although participants cannot be blinded to their own group allocation, they are blinded to the study’s hypothesis and to the treatment offered in the alternate group. Despite their different formats, both groups have parallel weekly sessions in terms of content and time, but at different locations and/or on different days. To minimize the risk of assessor’s unblinding, participants are asked (both in the consent form and at the time of each assessment) not to discuss their treatment with the independent assessor. In addition, in each center, the assessment is separated from the intervention session, by both time and location.

### Outcome measures and moderators/confounders

#### Primary outcome measure

The primary outcome measure is the mean percent reduction in the total number of UI episodes one year post randomization, as measured by a seven-day bladder diary [28]. For example, a participant experiencing four leaks per week pre intervention and two leaks per week at the one-year follow-up would be considered to have had a 50% reduction in leakage episodes. The number of leaks (as measured by the seven-day diary) is considered one of the most reliable measures of success for incontinence treatment and has been widely used in this type of research [16, 28, 44, 47, 51]. Moreover, the reduction in the number of daily leaks, expressed as a percentage, is information that can be easily understood by participants in pre-treatment counseling. As a measurement tool, the seven-day diary has a high compliance rate and good reproducibility [28, 56].

#### Secondary outcome measures

Several secondary outcomes are assessed in this study, in line with the recommendations of the 4th International Consultation on Incontinence [57] and the International Continence Society [58]. For each outcome, we have selected measurement instruments that have highest psychometric properties (validity, reliability, and response to change).

1. *The seven-day bladder diary:* The number of micturition per day/night is monitored to document lower urinary frequency during the day and nocturia [28].
2. *The 24-h pad test:* This is a validated measure of quantity of urine leakage in 24 h, as measured by the weight of the pads used during a 24-h period minus the weight of the pads before the test. It is identified as a realistic appraisal of the typical urine loss during ordinary daily activity [29]. The upper limit of “normal” for the 24-h pad test has been defined for continent women as being 1.3 g [47].
3. *The International Consultation on Incontinence Questionnaire (ICIQ) modules:* Five ICIQ modules, known to provide brief and robust measures of UI symptoms, quality of life, and outcome of treatment are used [32].
  i. *The ICIQ-Urinary Incontinence short form:* a four-item questionnaire which evaluates the impact of symptoms of incontinence on quality of life and outcome of treatment (0–21 overall score, with greater values indicating increased severity) [30].
  ii. *The ICIQ-Nocturia:* a two-item questionnaire which evaluates the impact of symptoms of nocturia on quality of life and outcome of treatment (0–8 overall score, with greater values indicating increased symptom severity) [31].
  iii. *The ICIQ-Vaginal Symptoms:* a 14-item questionnaire which evaluates the impact of vaginal symptoms and associated sexual matters on quality of life and outcome of treatment (0–53 vaginal symptoms subscale, 0–58 sexual matters subscale, 0–10 overall impact on quality of life subscale; with greater values indicating increasing problems) [33].
  iv. *The ICIQ-Female Lower Urinary Tract Symptoms sex:* a four-item questionnaire for evaluating sexual matters associated with female lower urinary tract symptoms (0–14 overall score, with greater values indicating increasing problems with sexual matters) [34].
  v. *The ICIQ-Lower Urinary Tract Symptoms quality of life:* a 20-item questionnaire which evaluates quality of life in urinary incontinent patients (0–14 overall score with greater values indicating increasing problems). The ICIQ-LUTSqol provides a detailed and robust measure to assess the impact of urinary incontinence on quality of life with particular reference to social effects [32].
4. *The geriatric self-efficacy index:* A 20-item instrument that enables measurement of whether a person is confident in their ability to prevent urine loss. It has been shown to be a reliable and valid instrument in the aging female population with UI [35].
5. *The Broom self-efficacy index (Part A):* A 14-item questionnaire to evaluate women's confidence in performing pelvic floor muscle exercise [36]. The Broom self-efficacy index has solid psychometric properties and is a useful tool to measure self-efficacy in doing PFM exercises [59].
6. *Patient global impression of improvement PGI-I:* a single-item global index used to measure improvement in urinary continence following PFMT on a 7-point scale that ranges from “very much better” to “very much worse.” The PGI-I has shown acceptable convergent and discriminant validity for measuring outcomes in studies of behavioral treatment for UI [37, 60].
7. *Satisfaction with treatment:* A single-item tool was used to document and capture perceived satisfaction with treatment: “satisfied” (does not need other treatments); “unsatisfied” (would like another treatment for UI) [17].
8. *PFM strength, morphometry and function:*
  i. *PFM strength on Oxford scale (digital palpation):* The Oxford scale is a 5-point scale used for PFM strength assessment amongst physiotherapists. Laycock and Jerwood have established intra-therapist reliability of this scale, tested in bent-knee lying [38, 61].
  ii. *PFM function:* An intravaginal dynamometric speculum, designed by members of our research team is used to measure passive (tone) and active forces (strength), speed of contraction, coordination with cough and endurance [39]. This instrument has been widely assessed for its psychometric properties including its reliability, validity, and responsiveness [39, 62].
  iii. *PFM Morphometry:* A Siemens Acuson Antares system with a 3–5-MHz curvilinear 3D/4D probe (in Montreal) and a GE Voluson Expert system with a 2–6-MHz curvilinear 3D/4D probe (in Sherbrooke) is used to evaluate several morphometric parameters at rest, during PFM contraction and on effort (cough and Valsalva): levator hiatus area and diameter, bladder neck position and displacement as well as levator plate height, using a validated and reliable methodology [40, 63, 64].
9. *The modified Dowel-Bryant Incontinence Cost index*

##### Costs related to interventions

The DBICI, a universally applicable questionnaire, has been adapted to measure the intervention costs for the two treatment groups [41, 65]. The validated DBICI has been used in RCTs with community-dwelling populations of women aged 40 years and over for non-surgical UI interventions [66] and is recommended by the International Consultation on Incontinence Research Guidelines [10, 42]. Section 1 of the DBICI documents, monthly self-reported personal incontinence expenditures (disposable and re-usable incontinence products), is used integrally. Section 2 deals with treatment expenditures and has been adapted as follows: (1) Treatment costs: rather than self-reported, treatment duration (in h) for each participant is based on statistics maintained by the research coordinators using a standardized form developed for a previous cost-effectiveness study [67]. The number of hours is multiplied by the mean hourly salary of the physiotherapists participating in the study. For the group-based PFMT, the total number of hours will be divided by the number (n = 8) of intended class participants (i.e. not actual attendance); (2) Other consultations: the frequency of visits to incontinence-related medical professionals is documented by category (general practitioners and specialists). A research assistant will phone participants at three months and six months post intervention in order to collect information. Estimated costs are based on the frequency and a mean cost per visit based on the public health system regulations for each category (Régie de l’assurance maladie du Québec). UI medication costs is excluded from the trial as those with UI medication are excluded at study entrance.

##### Pre-treatment covariates

In order to be able to adjust for potential imbalances, across the two randomization groups, in the distributions of covariates, that may be potentially associated with the outcomes, baseline measurement of the following variables will be carried out: basic socio-demographic data including age, BMI, general health status, medical history and medications, parity and obstetrical history as well as type and duration of UI. In secondary analyses, we will then use multivariable regression models to adjust the between-groups differences in the outcomes for these covariates, in addition to the pre-treatment values of the respective outcome variable.

##### Sample size calculation

Sample size was calculated so as to ensure the adequate power and type I error rate for testing the primary hypothesis of non-inferiority of the group-based intervention relative to the individualized intervention, in achieving the relative reduction (in %) in the number of UI episodes at one year. Sample size calculations followed CONSORT Guidelines for non-Inferiority trials [68]. First, based on clinical relevance [69] (minimum clinically relevant difference = 10%) and our pilot data [18–22], we set the “margin of equivalence” (i.e. the upper limit of the non-inferiority interval) as corresponding to a 10% difference between mean % reduction in the number of UI episodes in the “standard treatment” of the individualized intervention minus the group-based intervention arm. This implies that we test the null hypothesis H0:d ≤ 10%, where d denotes the true difference between the mean relative reductions in the number of UI episode in the two arms (individualized minus group-based) against the alternative hypothesis H1:d > 10%. According to the CONSORT guidelines, this choice of the “margin of equivalence” implies that the non-inferiority hypothesis should be rejected whenever the upper bound of the two-tailed (1 – 2α)% confidence interval (CI) for the difference between the two mean % reductions exceeds 10%. In the specific context of a non-inferiority trial, α is the selected risk of a false acceptance of the non-inferiority hypothesis (based on a one-tailed CI-based test, equivalent to an independent-group t-test); i.e. of a false conclusion that a truly inferior intervention is equally efficacious as the “standard treatment” [68].

To err on the conservative side, we relied on the “standard” 95% CI, which corresponds to a stringent one-tailed type I error rate of α = 0.025 (2α = 1–0.95 = 0.05). Similar to other recent non-Inferiority trials (e.g. [70, 71]) and CONSORT guidelines [68], for the purpose of sample size calculation, we assume that the true difference between the mean reductions (%) achieved by the two interventions will be zero (i.e. that they are equally effective). Note that this assumption of equivalence of the two interventions being compared in our trial is consistent with both (a) clinical expectations and (b) (limited) published evidence concerning young and middle-aged UI women [72–74]. Finally, both published trials, which evaluated similar interventions in older women, reported within-group standard deviations (SD) of the individual % reduction scores of about 27% [44, 75]. Accordingly, we assumed SD = 27% in our calculations.

Under the above assumptions, we estimated the sample size needed to ensure high (90%) power to demonstrate the non-inferiority of the group-based intervention (assuming, as mentioned above, that the true difference is 0%). Thus, we calculated N, for which the probability that the upper boundary of the two-tailed 95% CI for the difference in the mean relative reduction (Individual – Group) excludes the “upper threshold of non-inferiority” (10% difference), will reach at least 90%. The sample size calculations were performed using the program in the PASS software package, designed specifically for power/sample size estimation for non-inferiority trials [76]. Under the assumptions outlined above, we will need 155 participants per group, for a total of 310 individuals. We also need to account for possible losses to follow-up, as we expect a 15% attrition rate by the end of the one year, based on both our pilot data and similar published trials [16, 17, 22, 51]. Thus, we will recruit an additional 54 participants per group, increasing the total number of participants to 364 (364*(1–0.15) = 310).

#### Trial management

The PI and a research coordinator regularly contact (through emails, telephone, or in person) each participating urology and gynecology clinic in order to promote and monitor progress in recruitment. The PI, the evaluators, physiotherapists, research assistant, and the Sherbrooke and Montreal coordinators conduct conference calls or face-to-face meetings in order to monitor the study’s progress. All members of the research team are kept informed of progress through a newsletter, every six months.

All collected data are anonymized and kept under lock and key at Dr. Dumoulin and Morin’s laboratories at the research center of the Institut Universitaire de Gériatrie de Montréal and research center of the Cewntre Hospitalier de l’Université de Sherbrooke. After each assessment and on the same day, files are reviewed by the research assistant in order to identify missing data. Any missing information is retrieved immediately by research assistants directly from the study participants. Data are entered weekly – trimestrally (depending on recruitment rate) into a computerized database system *SPSS data Entry 4.0* both in Sherbrooke and in Montreal centers. The database is backed up on a weekly basis. A PIN number known only to the research group is required to access the data entry computer. A final quality-control step will be taken at the time of the data analysis by the trial statistician. Frequency distributions and ranges will be analyzed to detect outliers that could signal potential errors. The data will be analyzed without any nominative identifiers.

#### Adherence

Based on the results of our previous feasibility study and post-study focus group, no significant problems are expected with treatment adherence in either group [22, 26]. Indeed, participants (*n* = 27) in the feasibility study complied well with study demands in terms of attendance at the treatment sessions (90%), completion of the daily exercise program (78%), and data collection (95%) [26]. Post-study focus groups identified “close supervision by the physiotherapist” and a “short daily PFM exercise program” as key facilitators in the participant’s completion of a 12-week group-based PFMT program and their adherence to the daily home PFM exercise program [22]. Adherence will be measured as follows: the women will be provided with diaries to record home exercise adherence for the 12-week treatment session. In addition, exercise maintenance will be assessed with a standardized questionnaire, at three and six months post intervention as well as at the 12-month follow-up evaluation. Additionally, adherence to each supervised weekly treatment sessions will be recorded by the physiotherapists.

To ensure evaluation and treatment adherence, we used the following strategies: (1) individual consultations using vaginal digital palpation ensure that participants can perform a PFM contraction correctly (the physiotherapist is available before and after individual and group treatment sessions to provide tips and answer questions regarding the weekly treatment and home exercise program); (2) an exercise diary is given to each participant to bring home and is verified by the physiotherapist each week throughout the treatment; (3) the home exercise program comprises a short and simple daily PFM exercise program to facilitate exercise practice; (4) women in both groups are reminded of their appointments (evaluation and treatments) by a research assistant. They are scheduled at participants’ convenience; (5) follow-up contact calls are made at three and six months post intervention to maintain contact. Evaluators solicit information from participants on their intention to change address or telephone number and on the frequency of visits to incontinence-related medical professionals. They also verify if medications have been prescribed, surgery conducted, and if there are any other health problems likely to influence UI. Finally, they monitor their continued adherence to PFMT; and (6) participants are reimbursed for their travel and parking expenses related to participation in the study.

#### Data analysis

General analytical strategy: as opposed to conventional superiority trials, in non-inferiority trials per-protocol analysis is generally preferable to intention-to-treat (ITT) analysis, because the postulated hypothesis being tested assumes no difference between the two interventions [68]. Indeed, in non-inferiority trials, a “conservative” approach relies on a per-protocol analysis, which maximizes the probability of finding a statistically significant difference between the two groups, i.e. of rejecting the hypothesis that has motivated the trial. Moreover, the ITT approach includes all individuals who were initially randomized, regardless of their adherence, and thus may dilute, or even mask, the true difference between the interventions in the case of non-adherence or losses to follow-up [59]. In the extreme, if most participants do not adhere to the assigned intervention or fail to complete the follow-up assessment, the ITT analysis will automatically show no difference. To prevent such paradoxes, our main analysis will rely on the per-protocol approach, i.e. include only participants who will have completed the one-year assessment. On the other hand, because of its popularity, we will use the ITT approach in “sensitivity analyses,” as recommended by the CONSORT guidelines for non-inferiority trials [68]. Furthermore, our analyses will focus on (primary or secondary) outcomes at one year. Because all primary and secondary outcomes are measured on continuous scales, the methods outlined below for the analyses of the primary outcome will also apply to all secondary outcomes. However, the analysis of secondary outcomes will be exploratory in nature and some (e.g. for PFM function) may have more limited statistical power.

Preliminary descriptive analyses will compare the individuals in the two trial arms using means, medians, standard deviations, and interquartile ranges (IQR) for continuous variables and frequency distributions for categorical and binary variables. Any variable for which the difference between the two arms is considered clinically relevant will be adjusted for in the multivariable regression analyses (see below). The distribution of the outcome scores will be assessed for normality of residuals, using the Shapiro–Wilk test. In the case of significant violation of normality, appropriate parametric (e.g. logarithmic or Box-Cox) [77] or non-parametric [78] transformation will be applied, to meet the normality assumption underlying the univariate t-tests, as well as multivariable linear regression (see below).

Analyses for the primary outcome at one year (and secondary outcomes): The primary analysis will test our main hypothesis that group-based classes are not inferior to individualized treatment with respect to the primary outcome of relative (%) reduction in the number of leakages at one year post randomization. (For all outcomes, the analyses outlined below will be repeated using data on post-intervention assessment.) As recommended for non-inferiority trials, the hypothesis of non-inferiority will be accepted if, and only if, the upper bound of the two-tailed (1-2α) 95% CI (corresponding to a conservative Type I error of 0.025 for the one-tailed independent-group t-test) for the mean difference (in % reduction) excludes the non-inferiority threshold [68], set at 10% difference. This basic analysis will be extended to multivariable analyses. Specifically, two multivariable linear models, of increasing complexity, will be used to adjust the estimated difference between the % reduction at one year in the two groups for, respectively, (1) only the two stratification variables (center and type of UI), as well as the baseline number of UI episodes (to account for regression to the mean phenomenon); and (2) (if necessary) in addition to variables in model (1): any variable, for which a clinically important imbalance between the two arms is revealed by descriptive analyses (see above). In both models, the two-way interactions between the trial arm and (a) center and (b) UI type, will be tested using 1-df model-based F-tests to verify if the difference between the effects of the two interventions depend on either of those stratification variables. In the case of a statistically significant interaction (*p* < 0.05 for the F-test), the intervention effects will be estimated and tested separately in the respective subgroups [79]. The results of multivariable linear regression analyses will be summarized in terms of adjusted mean difference between the outcomes in the two trial arms, together with the 95% model-based CIs. In sensitivity analyses for primary and secondary outcomes at one year, ITT analyses will be conducted, using the same statistical methods.

#### Economic analysis

Mean, median, and interquartile (semi-IQ) will be used to describe the costs for the two groups. Cost-effectiveness analysis will be performed on the main outcome: reduction in number of leakages (%) one year after randomization and expressed as a ratio of incremental costs and percentage of leakages. In order to verify the robustness of the economic analysis, a sensitivity analysis will be conducted on the hypothesis that the costs for each participant should be between the 25th and 75th percentiles of the cost distribution.

#### Multivariable models to predict response to treatment

In additional analyses, multivariable logistic regression will be employed to assess patient characteristics associated with an increased likelihood of response to treatment, defined as at least 50% reduction in the number of UI episodes at one year. The logistic model will include, as independent variables, (a) the binary indicator of the trial arm, (b) the pre-intervention number of UI episodes, and (c) all previously listed covariates: age, BMI, general health status, level of physical activity, medical history and medications, parity and obstetrical history as well as type and duration of UI and current pad use. Independent, statistically significant predictors of response will be identified by *p* < 0.05 for the respective multivariable model-based two-tailed 1-df Wald test and their effects will be summarized by adjusted odds ratios with 95% CIs.

## Discussion

Considering demographic projections, human resources, and health system constraints, there is a pressing need to scale up evidence on the long-term benefits of low-cost interventions in urinary incontinent aging women and thereby improve clinical, functional, and social outcomes of this highly prevalent condition in this subpopulation. This GROUP trial, the first adequately powered RCT, seeks to study the non-inferiority of group PFMT in comparison to individual one-on-one PFMT in aging women with UI. If group-based PFMT classes prove to be equally effective, as well as more cost-effective, compared to individual one-on-one PFMT sessions, this trial could have a positive impact on the accessibility of continence care for aging women in Canada.

The results of this RCT will be relevant to clinicians and clinical decision-makers as well as to administrative stakeholders. It will have implications for the organization and the administration of continence care services. Ultimately, the results may influence the cost of treatment per individual, the accessibility of conservative management, and the use of more invasive UI interventions such as medication and surgery in older UI women.

### Dissemination of study finding

The results of this study will be disseminated through national and international scientific and professional conferences, in addition to undergraduate and postgraduate courses in PFM rehabilitation for physiotherapists.

## Trial status

This trial is actively recruiting participants (353/364). The trial is ongoing and has a planned duration of five years, with recruitment running from September 2012 to March 2017.

## Additional files

Additional file 1: Informed consent form. (PDF 82 kb)

Additional file 2: SPIRIT checklist. (DOC 120 kb)

## Abbreviations

DBICI: Dowell–Bryant Incontinence Cost Index
ICIQ modules: International Consultation on Incontinence questionnaire modules
ICIQ-FLUTSex: ICIQ-Female Lower Urinary Tract Symptoms sex
ICIQ-LUTSquol: ICIQ-Lower Urinary Tract Symptoms quality of life
ICIQ-N: ICIQ-Nocturia
ICIQ-UI short form: ICIQ-Urinary Incontinence short form
ICIQ-VS: ICIQ-Vaginal Symptoms
PFM: Pelvic floor muscle
PFMT: Pelvic floor muscle training
RCT: Randomized controlled trial
UI: Urinary incontinence
US: Ultrasound
