# Supplementary material for: Group physiotherapy compared to individual physiotherapy to treat urinary incontinence in aging women: study protocol for a randomized controlled trial
Source: Trials. 2017 Nov 16;18:544. doi: 10.1186/s13063-017-2261-4 (PMC5689182; doi:10.1186/s13063-017-2261-4)
Supplement: Supplementary file 1 — Informed consent form. (PDF 82 kb) [file 13063_2017_2261_MOESM1_ESM.pdf]

Institut universitaire de gériatrie de Montréal

INFORMED CONSENT FORM

Title of research project: Comparing two physiotherapies (pelvic-floor-muscle rehabilitation) to treat urinary incontinence in women aged 60 and over

Principal Investigator: Chantal Dumoulin, PhD, Research Centre of the IUGM.

Co-investigators:

- Mélanie Morin, PhD, Étienne-Le Bel Clinical Research Centre, CHUS.
- Dre Marie-Hélène Mayrand, MD, Centre Hospitalier de l'Université de Montréal.
- Michel Tousignant, PhD, Institut universitaire de gériatrie de Sherbrooke (IUGS)
- Michael Abrahamowicz, PhD, Statistician, McGill University

Research team: Valérie Elliott, Professional Research Associate

Funding Organisations : Canadian Institutes of Health Research (CIHR/IRSC)

Preamble

We would like to invite you to participate in a research project. However, before accepting to be a project participate and signing this informed consent form, please take time to read, understand and consider carefully the information below.

This form may contain words that you do not understand. We invite you to raise any questions that you deem pertinent with the lead project researcher or a member of her research team, including the clarification of any words or information about the project.

Nature and objectives of the research project

Urinary incontinence is one of the most common health problems in women aged 65 and older, affecting up to 55% of women living in the community, 20 to 25% suffer severe symptoms (more than 10 urinary incontinence episodes per week). Currently, physiotherapy treatments (pelvic-floor-muscle rehabilitation) are recommended as the first-line treatment for urinary incontinence in women. Different types of physiotherapy treatments exist; each has its advantages. To date, no study has compared the effectiveness of the two most-commonly utilised types of treatments for urinary incontinence in older women.

Thus, the objective of this study is to compare two types of physiotherapy treatments for urinary incontinence in women aged 60 and over and to establish the cost-effectiveness of each.

These two types of physiotherapy treatments, both of which are considered effective, consist of a program of pelvic-floor-muscle rehabilitation taught by a physiotherapist with relevant expertise in this field. The pelvic-floor-muscle rehabilitation program will be taught as a weekly one-hour course over 12 weeks.

To undertake this research project, we intend to recruit 440 participants, aged 60 and older, who will be divided into two study groups of 220 women.

Project procedures

The research will take place at the Research Centre of the Institut universitaire de gériatrie de Montréal and consist of four phases. The first phase includes eligibility and pre-treatment evaluation. Then, the second phase consists of 12 weeks of physiotherapy treatment for one hour each week. The third phase will consist of an end-of-treatment evaluation and, at 3- and 6-month intervals, telephone follow-up calls. Finally, the fourth phase will include an assessment one year after the start of your initial participation in the research project.

Phase I: Eligibility and pre-treatment evaluation

This phase will begin with a review of the informed consent form during which time you will have an opportunity to ask questions.

1. Eligibility assessment (15 minutes)

Afterwards, the physiotherapist, who is trained to conduct urinary-incontinence assessments, will collect your urinary diary and the soiled sanitary napkin(s), which you will have worn for 24 hours (referred to as a pad test). The urinary diary and sanitary napkins will be mailed to you a week before your appointment. You will also complete a questionnaire. This will allow us to confirm your eligibility for the study.

If you are eligible, you will be asked to proceed immediately to the pre-treatment evaluation. If you are not eligible, you will be given information materials on urinary incontinence, including a program of pelvic floor exercise.

2. Pre-treatment evaluation (1:45 hours)

The pre-treatment evaluation includes:

a) questionnaires (30 minutes)

The physiotherapist will ask you questions based on questionnaires that allow us to assess the severity of your urinary incontinence and its impact on your quality of life.

b) a physical assessment (5 minutes)

Your weight and height measurements will be recorded.

c) a vaginal assessment (20 minutes)

The physiotherapist will conduct a vaginal examination using manual palpation. This examination will enable us to teach you how to contract your pelvic floor muscles.

d) a dynamometric assessment (30 minutes)

You will be asked to undergo a dynamometric assessment. Pelvic floor dynamometry measures the forces produced by the pelvic floor muscles. Lying on your back with your knees bent, a dynamometer, which resembles a small speculum with two narrow branches (1.5cm), is inserted into your vagina to a depth of 5cm. When you perform a contraction, as if you are retaining urine, the force exerted by your muscles on the dynamometer is recorded via a computer and provides information on the tone, strength and endurance of your pelvic floor muscles.

e) a perineal ultrasound (20 minutes)

An ultrasound is an imaging technique that produces clear images of organs and the pelvic floor muscles. While lying on your back with your knees bent, a rounded probe connected to an ultrasound machine will be placed on your perineum, the area located between the vagina and anus. The physiotherapist will ask you to perform certain tasks such as contracting and relaxing your pelvic floor muscles. Images will be taken during these tasks.

Phase II: Exercise program to strengthen the pelvic floor muscles

The pre-treatment evaluation will be followed by 12 weeks of treatment, consisting of a pelvic-floor-muscles rehabilitation program taught by a different physiotherapist with expertise in the field.

Each session will include a 15-minute period devoted to urinary incontinence education and a 45-minute period devoted to pelvic-floor-muscle training, including strengthening, endurance, and coordination exercises as well as functional exercises (i.e., training the body for the activities performed in daily life) in different positions: on your back, on all-fours, sitting and standing. Alternative positions have been foreseen in order to accommodate individual limitations.

The two groups of women participating in the research project will each receive a different type of treatment. You will be assigned to one of the two groups. This assignment will be done at random (by chance).

Parallel to the treatment sessions with the physiotherapist, you will receive a program of exercises to do at home, 5 days per week (approximately 20 minutes per day).

Phase III: Post-treatment evaluation and follow-up telephone call

Remember, the physiotherapist who will be assessing you will not know which group you have been assigned to. It is important that this remain so throughout the post-treatment evaluation period.

1) Post-treatment evaluation (2 hours)

This evaluation, which will take place immediately post-treatment, is identical to the pre-treatment evaluation (except for the addition of questionnaires to evaluate your satisfaction) and will take place when you completed the 12-week treatment program. A second 7-day urinary diary and another pad test, again worn for 24 hours, must be returned during this evaluation.

2) Telephone follow-up (20 minutes by phone)

Telephone follow-up calls will be made at 3 and 6 months following the post-treatment evaluation to keep in contact with you: to verify if you intend to change address, are continuing to do the exercises, have changed or started taking medication, have had surgery, or have any health problems that may affect your urinary incontinence symptoms.

Phase IV: One-year follow-up evaluation (2 hours)

This evaluation will be conducted one year after the start of your participation in the project and will be identical to the post-treatment evaluation. You will also be asked to bring in a third 7-day urinary diary and a complete a third pad test, again, worn for 24 hours.

Project-related benefits

Although there can be no guarantees, you may derive a personal benefit from participating in this research project. Moreover, the results will contribute to the advancement of scientific knowledge in this field.

Risks associated with the research project

The risk of instruments becoming contaminated will be addressed through the application of the hygiene standards and infection-prevention controls in effect at the University Institute of Geriatrics of Montreal (Institut universitaire de gériatrie de Montréal: IUGM).

During the vaginal and dynamometric assessments, you may feel a slight discomfort. There is also the possibility that a slight vaginal discomfort and/or irritation may occur during or after these assessments.

During the perineal ultrasound, you may feel discomfort related to the slight pressure of the probe on the perineal area as well as a slight irritation after the examination.

During the teaching of the exercises, as well as their subsequent practice at home, there should be no problems except perhaps for some slight muscle aches.

Voluntary participation and the possibility of withdrawal

Your participation in this research project is voluntary. You can refuse to participate. You can also withdraw from the project at any time, without giving a reason, by making your decision known to the project's lead researcher or a member of the research team.

The researcher in charge of the research project, the IUGM Research Ethics Committee or granting-issuing agency may also terminate your participation without your consent, if new discoveries or information indicate that participation in the project is no longer in your best interest, if you do not comply with the instructions in the research project, or if there are administrative reasons to terminate the project.

If you withdraw or are withdrawn from the project, the information already obtained within the framework of this project will be kept as long as necessary to comply with regulatory requirements.

Any new information acquired throughout the course of the project, which could influence your decision to continue taking part in the project, will be communicated to you, without delay, verbally and in writing.

Confidentiality

Throughout your participation in this project, the lead researcher and her staff will collect and record information about you in a research file. Only the information required to meet the scientific objectives of this project will be collected.

This may include information regarding your past and present health, your lifestyle and the results of all tests, examinations and procedures undergone during this project. Your file may also include other information such as your name, gender, date of birth and ethnicity.

All of the information collected will remain, to the extent permitted by law, confidential. To protect your identity and privacy, you will be identified only by a numbered code. The code key, linking your name to

your research file, will be retained by the lead researcher.

The project researcher will use the data for research purposes in order to meet the scientific objectives of the project as described in this informed consent form.

Research data may be published in scientific journals or subject to scientific debate, but it will not be possible to identify you. Moreover, research data could be used for further data analysis related to the project or to develop future research projects. In addition, personal information, such as your name or address, will be kept by the lead researcher for 5 years after completion of the project; it will be destroyed thereafter.

For monitoring and control purposes, your research file can be accessed by a person authorized by the IUGM or its Research Ethics Committee (Comité d'éthique de la recherche de l'IUGM) or by a publically-mandated official. All these individuals and organizations adhere to a privacy policy.

In accordance with the law on access, you have the right to consult your file in order to check the information collected and amended it as warranted, and this, as long as the project researcher holds this information. However, to preserve the scientific integrity of the research, you may not have access to some of this information until your participation in the project has ended.

Subsequent studies

Do you agree that your research data be used for other research projects, be it in the fields of neuroscience and aging or in health promotion, care and intervention?

All research projects are evaluated and approved by the IUGM Research Ethics Committee (Comité d'éthique de la recherche de l'IUGM) before implementation. Your research data will be securely stored in the database of the IUGM Research Centre in compliance with its information-management policies. To protect your identity and the privacy of your research data, you will be identified only by a numbered code.

Your research data will be kept for as long as they are useful for the advancement of scientific knowledge. Once they cease to be useful, your research data will be destroyed. Further, note that at any time you may request the destruction of your research data by contacting the lead researcher for this project.

Do you consent to having your data used for other research under these conditions?
□ Yes □ No

Participation in further studies

Do you authorise the project researcher or a member of her team to re-establish contact with you to seek your participation in other research projects? Of course, during this call, you remain free to accept or refuse to participate in the proposed research. □ Yes □ No

Potential commercialization

Your participation in this research project could lead to the creation of commercial products. However, you are not eligible for any financial benefit.

Research project funding

The lead researcher of the project has received financial support from the identified funding agency (refer to page 1) to carry out this research project.

Compensation

In addition to the physiotherapy treatment, you will receive ten dollars ($10) for each evaluation, for a total of thirty dollars ($30), as compensation for your travel and participation in the research project. In addition, if you withdraw before the end of the project, you will receive a prorated compensation for your participation. Upon request, a parking ticket for the rear parking at the IUGM will be provided prior to each evaluation.

Damage compensation and the legal rights of research subjects

If you suffer any damage or injury whatsoever through your participation in this research project, you will receive the necessary care and services required by your state of health at no cost to you.

By agreeing to participate in this project, you do not waive any of your legal rights nor release the researchers, the funding agency and the establishment from their civil and professional liabilities.

Emergency medical procedures

Please note that the Institut universitaire de gériatrie de Montréal is not a short-term healthcare facility providing emergency services with an on-site presence of physicians 24/7. Consequently, in the event of a medical condition requiring immediate care, basic first aid will be provided by the staff on site and arrangements will be made to transfer you, if necessary, to the emergency services at a nearby hospital.

Resource personnel and access

If you have any questions about the research project or if you have a problem that you believe is related to your participation in the research project, you may contact the lead project researcher, Chantale Dumoulin PhD, at (514) 340-3540, ext 4153 or Mrs Valerie Elliott at (514) 340-3540, ext 4825.

For any questions regarding your legal rights as a subject participating in this research project or if you have any complaints or comments, you can contact the IUGM Commission of complaints and quality service at (514) 340-2109.

Monitoring ethical aspects of the research project

The IUGM Research Ethics Committee (Comité d'éthique de la recherche de l'IUGM) has approved and will monitor this research project. In addition, it will pre-approve any revision and/or amendment to the protocol and the informed consent form. For more information, please contact the Committee Secretariat by phone at (514) 340.2800, ext 3250 or by email: karima.bekhiti.iugm@ssss.gouv.qc.ca.

Consent

Title of research project: Comparing two physiotherapies (pelvic-floor-muscle rehabilitation) to treat urinary incontinence in women aged 60 and over

I. Participant's Consent

I have read the informed consent form. I acknowledge that the project has been explained to me, my questions answered and that I have been given adequate time to make my decision.

I consent to participate in this research project according to the conditions set forth in this document. A signed and dated copy of this informed consent form has been given to me.

____

Name and signature of the research subject Date

II. Signature of the person who obtained the consent if different from the researcher in charge of the research project.

I have explained the terms of the informed consent form to the research subject, and I have responded to all the questions posed by the subject.

____

Name and signature of the person obtaining the consent Date

III. Commitment and signature of the lead project researcher

I certify that the terms and conditions of the research contained in this informed consent form were explained to the research subject, and that all of the questions raised by the subject in this regard were answered and that I have clearly indicated to her that she is free to terminate her participation without prejudice.

I commit myself, along with my research team, to respecting the terms laid out in the informed consent form, and to returning a signed copy to the research subject.

____

Name and signature of the responsible researcher of the research project Date
